# Assessment of Ketamine and its Enantiomers in an Organophosphate-Based Rat Model for Features of Gulf War Illness

**DOI:** 10.3390/ijerph17134710

**Published:** 2020-06-30

**Authors:** Jackie Zhu, Elisa Hawkins, Kristin Phillips, Laxmikant S. Deshpande

**Affiliations:** 1 Department of Biology, College of Humanities & Sciences, Virginia Commonwealth University, Richmond, VA 23298, USA; zhujj@vcu.edu; 2 Departments of Neurology, School of Medicine, Virginia Commonwealth University, Richmond, VA 23298, USA; elisa.hawkins@vcuhealth.org; 3 School of Neuroscience, Virginia Tech, Blacksburg, VA 23298, USA; kfphill@vt.edu; 4 Pharmacology and Toxicology, School of Medicine, Virginia Commonwealth University, Richmond, VA 23298, USA

**Keywords:** organophosphates, DFP, depression, *R*-ketamine, *S*-ketamine, Sprague-Dawley rats

## Abstract

Approximately 33% of U.S. soldiers from the first Gulf War suffer from a multi-system disorder known as the Gulf War Illness (GWI). GW veterans suffer from a cluster of symptoms that prominently include fatigue and can include mood-related symptoms. Compared to traditional antidepressants, ketamine (KET) produces a fast-onset and long-lasting antidepressant response, but assessments of KET for GWI-related depression are lacking. The etiology of GWI is multi-factorial and exposure to organophosphates (OP) during deployment is one of the factors underlying GWI development. Here, male Sprague-Dawley rats were repeatedly exposed to an OP DFP and three months later these rats, when assessed on a battery of rodent behavioral assays, displayed signs consistent with aspects of GWI characteristics. When treated with a sub-anesthetic dose of KET (3, 5, or 10 mg/kg, i.p.), DFP-treated rats exhibited a significant improvement in immobility time, open-arm exploration, and sucrose consumption as early as 1 h and much of these effects persisted at 24-h post-KET injection. KET’s stereoisomers, *R*-KET and *S*-KET, also exhibited such effects in DFP rats, with *R*-KET being the more potent isomer. Our studies provide a starting point for further assessment of KET for GWI depression.

## 1. Introduction

Approximately one-third of nearly 700,000 U.S. soldiers deployed during the first Gulf War (GW) suffer from a chronic multi-system disorder known as Gulf War Illness (GWI). A definition of GWI was initially proposed by Dr. Steele [1], which was subsequently endorsed by The National Academy of Medicine (formerly The Institute of Medicine). Accordingly, GWI is defined as the presence of a spectrum of chronic symptoms in GW service members that persist for 6 months or longer in at least two of six categories: development of fatigue, mood and cognitive changes, musculoskeletal changes, gastrointestinal symptoms, respiratory, and neurological disorders [2]. In particular, veterans with GWI have reported cognitive dysfunction and increases in mood disorders including anxiety and depression [3]. 

There are multiple factors to which the development of GWI could be potentially attributed, some of which may serve as confounders in assessing the role of others as causes of GWI. Deployment complexities led to soldiers being exposed to a variety of pharmaceuticals, chemicals, and environmental factors. The Institute of Medicine, The Research Advisory Committee on Gulf War Illness, and other researchers have studied the effects of many deployment-related factors such as sustained exposure to insecticides and pesticides, ingestion of pyridostigmine bromide pills, inhalation of dust particulate matter and oil-well fire smog, and exposure to depleted uranium, as probable causes for GWI development [2,4,5,6]. Following the careful evaluation of these studies, exposure to organophosphates (OP) has been strongly implicated as one of the major factors for GWI [2,6,7,8,9]. The OP category of compounds includes commonly used pesticides and lethal nerve agents. More than 35 types of pesticides and pesticide products were used by military personnel during the first GW out of which Office of the Special Assistant for Gulf War Illnesses considers 12 pesticides of concern either because of toxicity or expected exposure [6,7,10,11]. Further, exposure to OP nerve gas sarin [12,13] is also well documented during the first GW. There are also series of in-depth studies conducted in civilian population that provides strong evidence in favor of a causal relationship between OP exposure and significant neuropsychiatric disorder including mood destabilization and cognitive impairment [14,15,16,17], a condition that shares GWI-like features seen in these OP survivors. While significant advances have been made in developing treatments for acute OP toxicity [18,19,20], little is known about the mechanisms that cause chronic morbidity in survivors of OP/nerve agent exposure, and little research has focused on curing these morbidities. Thus, despite treatment recommendations, GWI veterans continue to suffer from these morbidities that dramatically affect their well-being and productivity [2].

Studies have shown that depression is a persistent GWI neurological symptom and more prevalent in deployed first GW veterans than their non-deployed counterparts [3,21,22]. Meta-analyses and systematic review showed that GW veterans were more than twice as likely to experience depression compared with military personnel who were not deployed to the first GW [22]. The current treatment recommendations from the Institute of Medicine for GWI depression include antidepressant medications and psychotherapy such as cognitive behavioral therapy [2,23]. However, treatment with traditional antidepressants is limited by a delay in the onset of therapeutic effects [24], and many patients do not achieve sustained remission of depressive symptoms [25]. In fact, one-third of patients with major depressive disorder are considered to have treatment-resistant depression [26]. Thus, there is a need to identify faster-onset antidepressants for the treatment of both depression and GWI-related depression.

The N-methyl-D-aspartate receptor (NMDAR) antagonist ketamine (KET) is reported to produce a rapid-onset and sustained antidepressant response [27]. The clinical antidepressant effects of KET were first reported over a decade ago [28]. A single intravenous infusion of sub-anesthetic dose of KET produced rapid antidepressant effect (within hours) that was sustained for up to a week even in patients with refractory depression [29]. Recently, the United States Food and Drug Administration (U.S.FDA) approved a nasal spray of (S)-ketamine, an enantiomer of ketamine, for treatment-resistant depression [30,31]. However, there are no studies to date that have determined whether KET or its enantiomers could be effective in treating GWI-associated depression.

We have developed a rat model that recapitulates some aspects of GWI related neurological signs such as the mood and memory dysfunction that are reported by GW veterans. In this model, rats are administered multiple low-doses of a nerve agent surrogate with an OP agent diisopropyl fluorophosphate (DFP) in an attempt to mimic GW-related OP exposures. Weeks after these initial exposures, DFP rats displayed signs on rodent behavioral assays that reflected aspects of chronic depression, anxiety, and memory impairment [32]. These DFP rats also exhibit protracted neuronal calcium elevations and hippocampal injury [32]. This pre-clinical OP-based rat model has been helpful in identifying mechanisms underlying the development of GWI and has been used to screen drugs that could prove to be effective therapies for treatment of GWI [33]. In this study, we utilized this DFP-based rat model for GWI-like neurological morbidities to investigate whether treatment with racemic KET (*R, S* KET) or its individual enantiomers (*R*-KET, *S*-KET) could produce the same reported rapid-onset and long-lasting antidepressant-like effects following GW-relevant OP exposure. 

## 2. Materials and Methods

### 2.1. Animals

All animal use procedures were in strict accordance with the National Institute of Health (NIH) Guide for the Care and Use of Laboratory Animals. The experimental protocol was approved by Virginia Commonwealth University’s (VCU) Institutional Animal Care and Use Committee (IACUC) and by the Animal Care and Use Review Office (ACURO) of the United States Army Medical Research and Material Command (USAMRMC). Male Sprague-Dawley rats (Envigo, Indianapolis, IN, USA) weighing ~300 g and 9 weeks of age were used in this study. Animals were housed two per cage at 20–22 °C ambient temperature with a 12-h light–dark cycle (lights on 0600-1800 h) and given free access to food and water. Animals were acclimated to the vivarium conditions for one-week period before DFP exposure.

### 2.2. Chemicals

Racemic Ketamine (Zetamine^®^) in injectable form (VetOne) was obtained from VCU Division of Animal Resources Pharmacy. *R*-ketamine and *S*-ketamine were obtained from Tocris Bioscience (Bio-techne Corp, Minneapolis, MN, USA). DFP and other chemicals were obtained from Millipore-Sigma (St. Louis, MO, USA). All the drugs, dilutions, and buffer solutions were made fresh daily.

### 2.3. DFP Exposure 

DFP exposures were carried out as described previously [32,33,34]. Briefly, rats were injected with DFP (0.5 mg/kg) once daily for 5 days, while control rats received DFP vehicle injections (PBS) for the same period. DFP was injected subcutaneously (s.c.) on the back of the rat with a volume ranging between 0.30 and 0.32 mL. Animal health including weight measurements was assessed daily during the exposure and for an additional seven days following DFP injections. A small percentage of DFP injected rats (less than 5%) exhibited mild tremors around the 5th day of dosing that resolved within 24 h without the need for any pharmacological intervention. DFP-exposed rats showed no observable seizures or other hyper-cholinergic activity acutely or chronically. Rats were subsequently monitored once weekly for general health until their use in experimental studies.

### 2.4. Experimental Design

**Figure 1:**
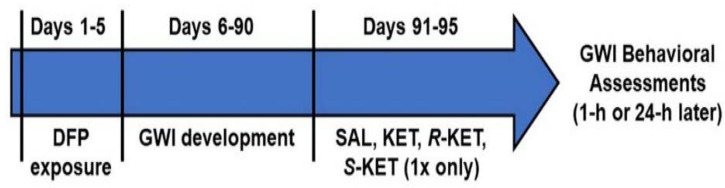
**Experimental Design** Nine-week old male Sprague-Dawley rats were exposed once-daily to organophosphates (OP) agent DFP (0.5 mg/kg, s.c.) or vehicle (ice-cold PBS). About three-months later, diisopropyl fluorophosphate (DFP) rats were treated with saline (SAL) or various doses of either ketamine (KET) or its enantiomers: *R*-ketamine (*R*-KET) or *S*-ketamine (*S*-KET) and assessed for behavioral effects at 1 or 24 h post-injection.

At 3 months following DFP exposures, no significant differences in weights were observed between control and DFP-exposed rats. These rats were subjected to a battery of behavioral tests in a quiet dimly lit room to assess for signs of depression. Separate cohorts of DFP-treated and saline-treated rats were then randomized into four main groups based on the treatment they received: Saline (SAL), KET, *R*-KET, *S*-KET. Within each group, rats were further divided into sub-groups based on the dose and the time of behavioral assessment. Only one behavioral assay was carried out each day (Figure 1).

### 2.5. Open Field Test (OFT)

An OFT was used to assess the effect of drug treatment on general activity levels and gross locomotor activity [35]. OFT outcome could be affected by fatigue experienced by the animal. Indeed, OPs are reported to produce fatigue [36], possibly through their effect on mitochondrial impairment [37]. GW veterans also report chronic fatigue and mitochondrial dysfunction in GWI has also been demonstrated [38].

Briefly, rats were placed in a black Perspex box (90 cm L× 60 cm W × 50 cm H) in a quiet, dimly illuminated room for 10-min. Distance travelled during the exploration period was automatically assessed using an overhead camera and a video-tracking system (Noldus Ethovision XT 11). The arena was cleaned with a 70% ethanol solution and dried completely in between each subject to eliminate any potential odor cues.

### 2.6. Forced Swim Test (FST)

Porsolt’s modified FST was used to assess signs of behavioral despair, as indicated by the amount of time an animals stays immobile during the test session [32,34,35,39]. There are several factors that influence FST outcome [40] and FST in itself can affect immune and endocrine changes thereby affecting interpretation of treatment outcomes [41]. An increase in FST immobility time is often construed as an indication of despair and a decrease in FST immobility time following pharmacological intervention is regarded as the drug possessing a potential antidepressant profile. However, it should be mentioned that alterations in the immobility times could merely reflect the fatigue experienced by the animal on this test considering their previous OP exposure [36,37,38].

Briefly, animals were placed in a glass cylindrical chamber (46 cm H × 30 cm D) filled with water (30 cm height, 25 °C) forced to swim for 6 min. Swimming sessions were recorded for off-line analysis. Active (swimming, climbing, diving) and passive (immobility) behavior was evaluated by two reviewers blinded to the treatment conditions. Immobility (primary outcome) was defined as the period during which the animal floats in the water making only those movements necessary to keep its head above water. The tank was emptied and thoroughly cleaned between sessions.

### 2.7. Sucrose Preference Test (SPT)

This test assesses signs of anhedonia-like behavior by monitoring a rat’s preference to sucrose-laced water [32,34,35,39]. An increase in the consumption of sucrose-water over plain-water is interpreted as an animal’s preference for pleasure-seeking (hedonic) behavior. Several factors can affect SPT behavior including concentration of sucrose, circadian rhythms, side-bias, and weight of the animals [42]. While we control for these variables when implementing an SPT paradigm, the possibility of OP-induced changes in the metabolic state of the animal, insulin resistance, and oxidative stress could affect SPT outcome.

Briefly, rats were habituated to having two bottles in the cage lid for three days. The bottles were fitted with ball-bearing sipper tubes that prevented fluids from leaking. Following this acclimation, rats were deprived of water but not food for a 20-h period. At the end of this period, two bottles were introduced in the cage lid and rats had a free choice of either drinking the 1% sucrose-laced solution or plain water over the next 24-h period. The positions of two bottles were switched mid-way to reduce any confounding effects produced by a side bias. Sucrose preference was calculated as a percentage of the volume of sucrose intake over the total volume of fluid intake and averaged over the testing period.

### 2.8. Elevated Plus Maze (EPM)

This test assesses for signs of anxiety by considering the innate behavior of rats to prefer dark enclosed spaces over bright open spaces. An increase in open arm activity (duration and/or entries) reflects anti-anxiety behavior [32,34,35,39,43]. Like FST, EPM behavior could also be affected by baseline motor activity and fatigue experienced by the animal due to previous OP exposure [36,37,38].

The maze (Med Associates Inc., St. Albans, VT) was made of black polyvinyl chloride and consisted of four arms, 50 cm long × 10 cm wide, connected by a central square of 10 × 10 cm: two open without walls and two closed by 31-cm-high walls. All arms were attached to sturdy metal legs; the maze was elevated 55 cm above the floor level and was set in a dimly lit room. A video camera was suspended above the maze to record the rat movements for analysis. A video-tracking system (Noldus Ethovision XT 11) was used to automatically collect behavioral data. The procedure consisted of placing the rats at the junction of the open and closed arms, the center of the maze, facing the open arm opposite to where the experimenter was. The video-tracking system was started after the animal was placed in the maze so that the behavior of each animal was consistently recorded for 5 min. At the end of the 5 min test session, the rat was removed from the plus maze and returned to its home cage. The maze was cleaned with 70% ethanol and air-dried to remove any scent traces and allowed to dry completely before introducing the next animal in the arena. The time spent and entries made in the various arms of EPM were calculated.

### 2.9. Data Analysis

Data were analyzed and graphs plotted using the SigmaPlot 14 software (SPSS Inc, Chicago, IL, USA). For comparing differences between SAL-treated GWI rats vs. KET-treated GWI rats on the outcomes of behavioral studies, we used a two-sided *t*-test, or a One-Way ANOVA followed by a post-hoc Tukey’s test wherever appropriate. In all cases, statistical significance was indicated by * *p* < 0.05.

## 3. Results

### 3.1. Chronic Effects of Repeated, Low-Dose DFP Exposure on Rat Behavior

**Figure 2:**
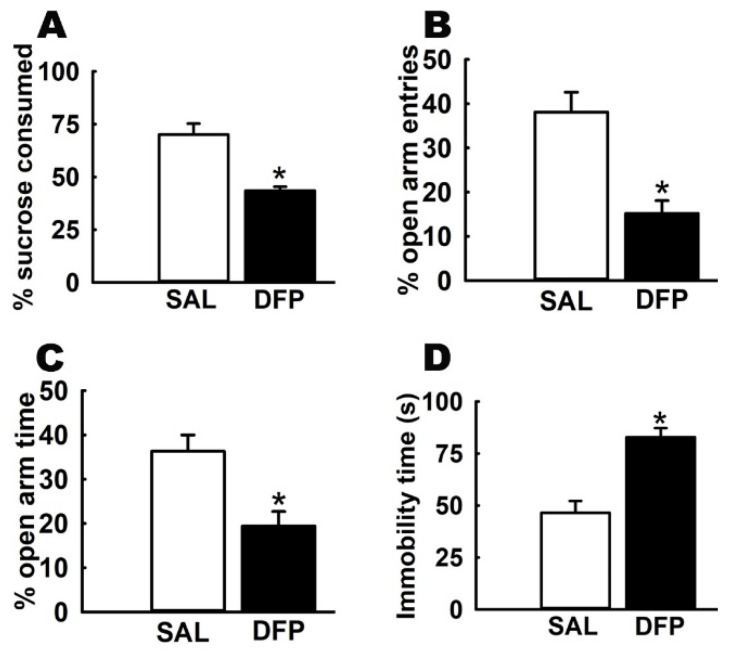
**Repeated, low-dose DFP-treated rats exhibit signs of a depression-like condition.** DFP exposure (0.5 mg/kg, s.c., 1× daily for 5 days) produced significant alterations in rat behavior when assessed at 3 months following exposure, compared to saline-treated age-matched control rats. (**A**) SPT: decreased sucrose consumption. (**B**,**C**) EPM: decreased entries and time-spent in the open-arm of EPM (**D**) FST: increased immobility time (data expressed as mean ± SEM, * *p* < 0.05, *t*-test, n = 6 rats/test).

At 3-months post-DFP-exposure, age-matched saline-treated control rats and DFP-treated rats were subjected to a battery of rodent behavioral assays. On the SPT, DFP rats consumed 43.5 ± 1.9% of sucrose-laced water, which was significantly lower than the 70.1 ± 5.2% of sucrose preference exhibited by control rats (Figure 2A). On the open-arm of the EPM, DFP rats spent 19.4 ± 3.3% time and made 15.2 ± 2.9 entries, which was significantly lower than the open-arm time of 36.3 ± 3.7% and 38.1 ± 4.5% of open-arm entries made by control rats (Figure 2B,C). On the FST, DFP rats exhibited an immobility time of 82.8 ± 4.4 s, which was significantly higher than the immobility time of 46.5 ± 5.7 s exhibited by control rats (Figure 2D, n = six rats/test, *t*-test, * *p* < 0.05). In agreement with our previous studies [32,33,34], the increased FST immobility time, lowered exploration of the open-arm on the EPM, and decreased sucrose consumption on the SPT in DFP-treated rats confirmed the presence of signs of a depression-like state following OP exposure. Separate cohorts of such DFP-treated rats were used for assessment of the effects of KET and its enantiomers on GWI-like depressive signs.

### 3.2. Effects of KET on Locomotor Behavior in GWI Rats

**Figure 3:**
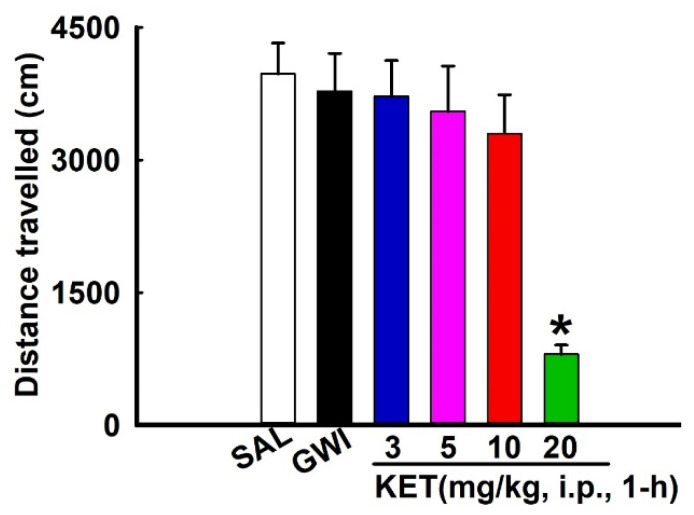
Dose-dependent effect of ketamine (KET) on the OFT in a DFP rat model for GWI. Baseline locomotor activity in DFP-treated rats (GWI) was not different compared to saline-treated, age-matched control rats (SAL). KET (3-, 5-, 10 mg/kg, i.p.) exhibited no significant differences in the distance traveled on the OFT compared to saline-treated GWI rats when tested at 1 h post-injection. KET (20 mg/kg, i.p., 1-h) significantly reduced the distance travelled on an OFT compared to GWI rats (Data expressed as mean ± SEM, * *p* < 0.05, One-way ANOVA, Tukey test, n = 5 rats/group).

As mentioned earlier, OPs are reported to produce fatigue [36], which could affect outcomes on the behavioral tasks used in this study. We used OFT to assess the effect of drug treatment on locomotor activity. As shown in Figure 3, compared to saline-treated age-matched control rats (SAL), DFP-exposed rats (GWI) were not significantly different, suggesting that baseline locomotor activity was not affected following previous DFP exposure.

Preclinical studies in rodents have reported KET being used between a 5–20 mg/kg range. KET is a known anesthetic, and we therefore first investigated a dose-dependent effect of KET on locomotor activity using the OFT. As shown in Figure 3, KET at 3-, 5-, 10 mg/kg, i.p. had no significant effect on the distance travelled in the open-field apparatus compared to saline-treated DFP rats when tested at 1 h after injection. In contrast, KET (20 mg/kg, i.p., 1-h) produced major motor incoordination and significantly reduced the distance travelled on an OFT compared to DFP rats (n = five rats/dose, One-Way ANOVA, Tukey-test, * *p* < 0.05).

### 3.3. Acute Effect of KET on FST Behavior in GWI Rats

**Figure 4:**
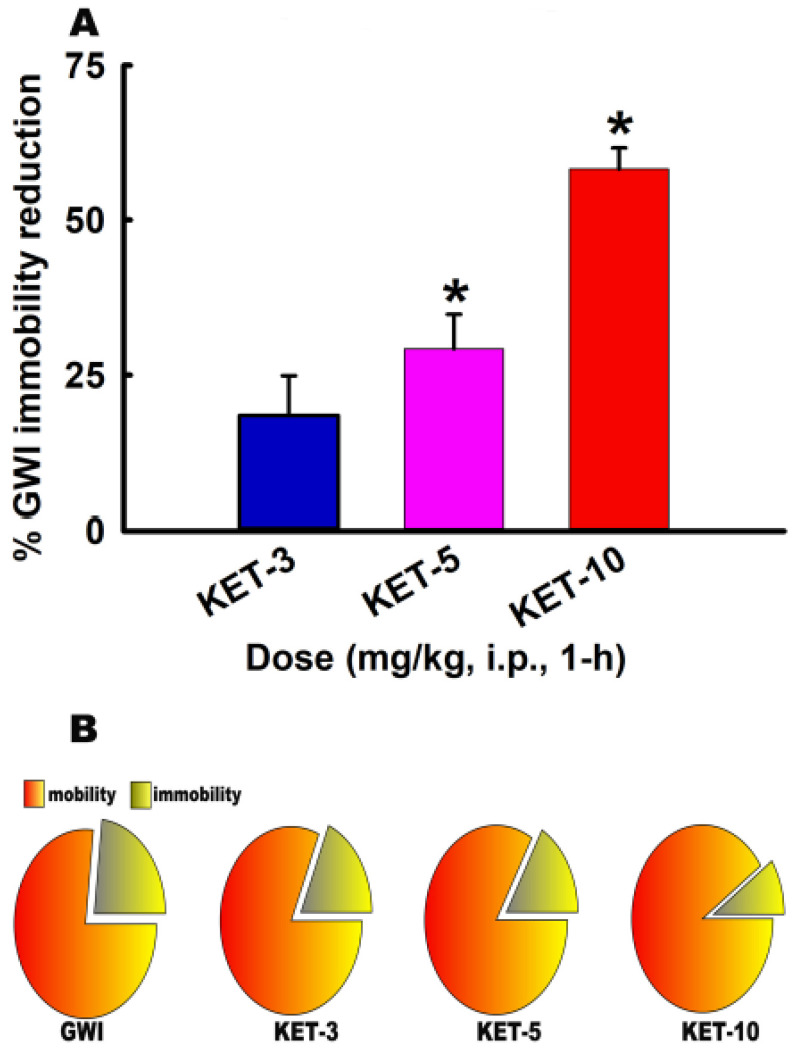
Rapid effect of KET on the forced swim test (FST) immobility in a DFP rat model for Gulf War Illness (GWI) (**A**) KET (5 and 10 mg/kg, i.p.) but not 3 mg/kg produced significant reductions in immobility times compared to GWI rats when tested at 1 h post-injection (data expressed as mean ± SEM of percent reduction in GWI immobility, * *p* < 0.05, One-way ANOVA, Tukey test, n = 7 rats/group). (**B**) Pie-chart depicting relative mobility vs. immobility percentages (exploded slice) for GWI rats treated with saline or various doses of KET.

Based on OFT data, we investigated the effects of KET doses that did not affect motor function. Thus, GWI rats were treated with KET at one of the three low, sub-anesthetic doses (3, 5, and 10 mg/kg, i.p.) and subjected to FST 1-h later. KET decreased immobility time dose-dependently on the FST. As shown in Figure 4, KET (3 mg/kg) caused an 18% reduction in FST immobility time while KET (5 mg/kg) and KET (10 mg/kg) produced even greater reductions in FST immobility times, 29% and 58% respectively. Only the KET 5- and 10-mg/kg doses produced significant reductions in immobility times, and while KET-3 mg/kg also produced a reduction in immobility time, the percent reduction was not significantly different than with saline-treated GWI rats (n = seven rats/dose, One-Way ANOVA, Tukey-test, * *p* < 0.05).

### 3.4. Sustained Effect of KET on FST Behavior in GWI Rats

**Figure 5:**
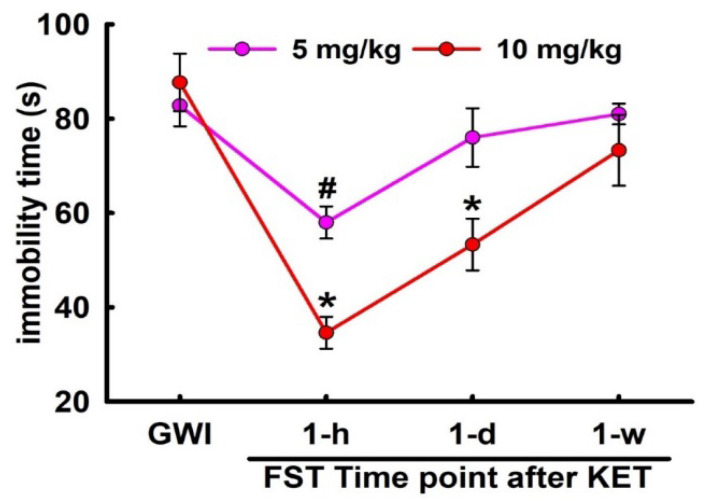
Sustained effect of KET on the FST behavior in a DFP rat model for GWI. GWI rats were treated with effective doses of KET (5 and 10 mg/kg, i.p.) and subjected to FST session at 1 day and 1 week after KET injection. Significant long-lasting decreases in immobility time were observed only with KET (10 mg/kg) at 1 day but not at 1 week (data expressed as mean ± SEM GWI immobility, * *p* < 0.05, *t*-test, n = 7 rats/group).

Based on the acute FST data, a separate cohort of GWI rats were treated with effective doses of KET (5 and 10 mg/kg, i.p.) and subjected to FST session at 1 day and 1 week after KET injection to investigate the persistence of KET’s acute effects. As shown in Figure 5, both the doses of KET at 1 h exhibited significant reductions in immobility time compared to saline-treated GWI controls. At 1-day post-KET-treatment, significant decreases in FST immobility time were observed only with KET (10 mg/kg) but not with KET (5 mg/kg). However, the sustained effects of KET (10 mg/kg) were no longer observed at 1 week post-KET-administration and the FST immobility time, while lower than saline-treated GWI rats, was not significantly different (n = seven rats/dose, *t*-test compared to GWI immobility, * *p* < 0.05).

### 3.5. Effect of KET on Sucrose Comsumption in GWI Rats

**Figure 6:**
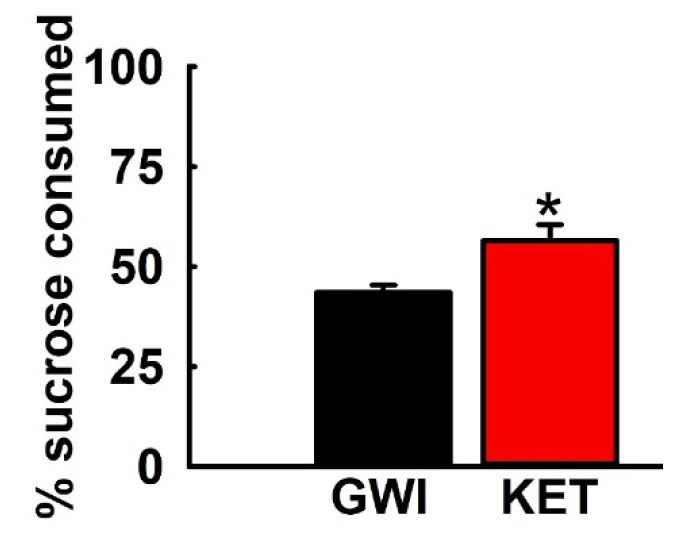
Effect of KET on the SPT in a DFP rat model for GWI. GWI rats treated with KET (10 mg/kg, i.p.) had a significantly higher sucrose preference compared to saline-treated GWI rats (data expressed as mean ± SEM % sucrose intake over a 24 h period, * *p* < 0.05, *t*-test, n = 6 rats/group).

Based on the FST data, a separate cohort of GWI rats were treated with the most effective dose of KET (10 mg/kg, i.p.) and analyzed for fluid consumption patterns on a SPT. As shown in Figure 6, KET-treated GWI rats displayed increased sucrose preference compared to saline-treated GWI rats. Thus, KET-treated GWI rats exhibited a sucrose preference of 56.5 ± 4.04%, which was significantly greater that the sucrose preference of 43.5 ± 1.9% exhibited by saline-treated GWI rats (n = six rats/group, *t*-test, * *p* < 0.05).

### 3.6. Acute Effect of R-KET and S-KET on FST Behavior in GWI Rats

**Figure 7:**
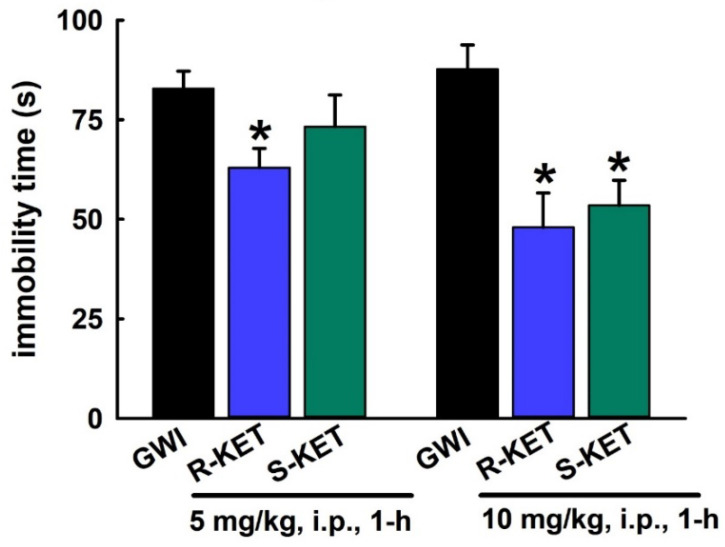
Rapid effect of *R*-KET and *S*-KET on the FST behavior in a DFP rat model for GWI. On the FST, both *R*-KET and *S*-KET (10 mg/kg, i.p.) produced reductions in FST immobility time in GWI rats when tested at 1 h post-injection. At the 5 mg/kg dose, only *R*-KET produced a significant reduction in FST immobility time (data expressed as mean ± SEM immobility, * *p* < 0.05, *t*-test, compared to GWI immobility, n = 7 rats/group).

KET is a racemic mixture consisting of equal parts *R*-KET and *S*-KET. We first investigated the acute FST effect of KET enantiomers using the same effective doses that were found to produce a significant effect for the KET racemic mixture (Figure 4). Thus, the dose-dependent rapid FST effects of KET enantiomers (5 and 10 mg/kg, i.p., 1 h) were tested using FST. As shown in Figure 7, both *R*-KET and *S*-KET (10 mg/kg, i.p.) produced significant reductions in FST immobility time compared to saline-treated GWI rats (87.7 ± 6.1 s vs. 48 ± 8.6 s and 53.5 ± 6.3 s), respectively. *R*-KET appeared to be more potent than *S*-KET, since, at the lower 5 mg/kg dose, only R-KET produced a significant reduction in FST immobility time while *S*-KET-mediated reductions in FST immobility times were not significantly different compared to saline-treated GWI rats (n = 7 rats/ dose, *t*-test compared to GWI immobility, * *p* < 0.05).

### 3.7. Sustained Effect of R-KET and S-KET on FST Behavior in GWI Rats

**Figure 8:**
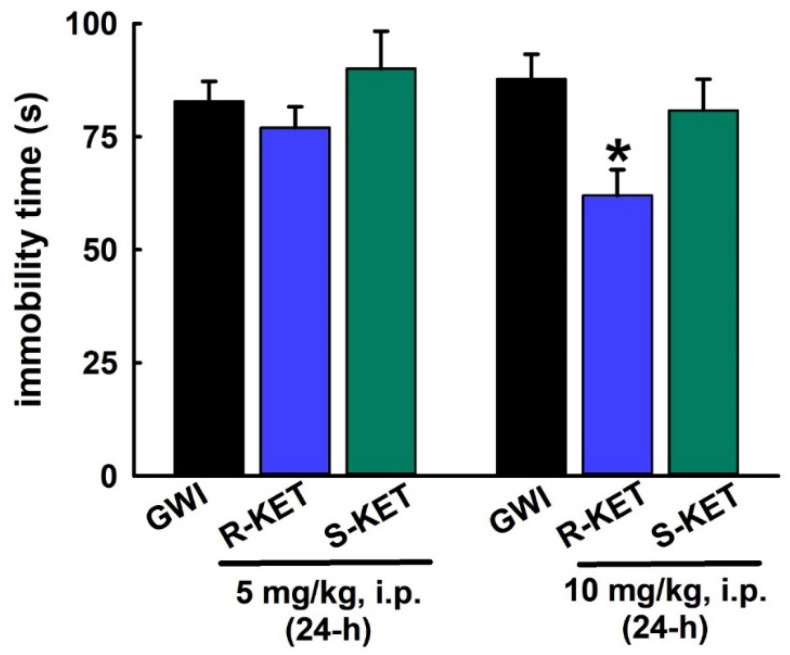
Sustained effect of *R*-KET and *S*-KET on the FST behavior in a DFP rat model for GWI Separate cohorts of GWI rats were tested on FST at 24 h post-injection. The 5 mg/kg dose of *R*-KET and *S*-KET were not effective in exhibiting significant reductions in the immobility times at 24 h post-injection. Only *R*-KET (10 mg/kg, i.p.) produced a significant reduction in the immobility time at 24 h (data expressed as mean ± SEM immobility, * *p* < 0.05, *t*-test, compared to GWI immobility, n = 7 rats/group).

Like KET, we next investigated the long-lasting effects of both *S*-KET and *R*-KET on FST behavior in a separate cohort of GWI rats using the same effective doses (5 and 10 mg/kg, i.p.). As shown in Figure 8, when tested at 24 h post-injection, the 5 mg/kg dose was not effective in significantly lowering FST immobility times for both *S*-KET and *R*-KET. Only *R*-KET (10 mg/kg, i.p.) produced a sustained FST effect at 24 h, as evidenced by significant reductions in immobility time compared to saline-treated GWI rats (87.7 ± 5.5 s vs. 62 ± 5.7 s). Like the acute FST effects, *R*-KET appeared to be more potent at affecting FST behavior than *S*-KET (n = seven rats/dose, *t*-test compared to GWI immobility, * *p* < 0.05).

### 3.8. Effects of KET, R-KET, S-KET on EPM Performance in GWI Rats

**Figure 9:**
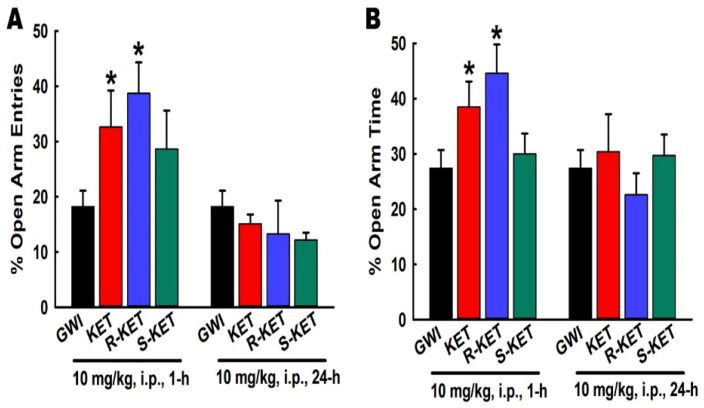
Open-Arm behavior on an EPM. KET (10 mg/kg. i.p.) and R-KET (10 mg/kg, i.p.) at 1 h after injection exhibited significant increase in the percent of open-arm entries (**A**) and time spent exploring the open-arm (**B**) compared to saline-treated GWI rats but, this effect was lost by 24 h. S-KET was not able to affect open-arm behavior at either timepoint. (Data are expressed as mean ± SEM, * *p* < 0.05, One-Way ANOVA, Tukey Test, compared to GWI, n = 7 rats/group).

Based on the previous behavioral assessments, the 10 mg/kg dose was found to be most effective at both the 1- and 24-h timepoint. We used this dose to assess the effect of KET and its enantiomers on EPM behavior (Figure 9). At 1 h post-injection, compared to saline-treated GWI rats, both KET and R-KET produced a significant increase in the number of entries and time spent in the open arm of EPM. However, S-KET did not affect open-arm behavior on the EPM. At 24 h post-injection, the effects of KET and R-KET could no longer be observed, while S-KET treatment remained ineffective in producing any significant changes in the EPM behavior (n = six rats/ treatment group/timepoint, one-way ANOVA, Tukey test, * *p* < 0.05).

## 4. Discussion

Exposure to OP agents in rodents either alone or in combination with other GW-deployment-related factors has been demonstrated to produce a myriad of central and peripheral signs that are commonly reported by GWI veterans [2,6]. Amongst these symptoms, GW service members increasingly report mood disorders, and these neurological morbidities have been difficult to manage with existing therapies [22,44,45]. The recent approval of KET by the US-FDA [30,31] is considered as a major development in psychotherapy owing to its unique ability to produce a fast-acting and a long-lasting antidepressant action [29]. KET’s rapid onset is considered to be ideal in depression patients with risk of suicide [46], compared to traditional antidepressants which have delayed onset of therapeutic effects, and KET infusions have been reported to decrease suicidal ideations within 24 h [47]. It is therefore interesting to investigate whether KET could extend similar a robust antidepressant effect in a depression phenotype that has been proven difficult to manage with current therapies.

Depression is a complex psychological phenomenon, and as such is difficult to assess in rodents using a single behavioral test [35,39]. We therefore used a combination of behavioral assays that measure certain aspects of signs of depression that are reported in humans. In agreement with our previous studies [32,33,34], DFP-exposed rats exhibited significantly higher immobility time in the FST, did not show higher preference towards sweetened water in the SPT, and exhibited lowered exploration in the open-arm of the EPM. Taken together, these behaviors suggest that repeated low-dose DFP exposure produces chronic neuropsychiatric signs in rodents. This model is therefore useful to assess certain features of GWI following OP-only exposures.

Experimental outcomes on rodent behavioral tasks, particularly the ones used in this study (OFT, FST, EPM), are essentially dependent on the locomotor function. Any changes in the baseline motor function due to experimental variables or paradigms could influence behavioral patterns and, correspondingly, the interpretation of the data. Indeed, OPs are reported to produce fatigue [36], possibly through their effect on mitochondrial impairment [37]. GW veterans also report chronic fatigue [1,2], and mitochondrial dysfunction in GWI has also been reported [38]. While our OFT data indicated that baseline locomotor activity was not different in DFP-exposed rats, stress experienced by DFP rats on the FST or the EPM could influence these fatigue-related behaviors. As far as the SPT outcomes are concerned, the basal glucose level in DFP rats could be a potential confounding factor. We did not measure blood glucose in our DFP-treated rats, but it has been shown that the oxidative stress and mitochondrial damage that are seen following OP exposures [37,38] are also risk factors for insulin resistance which could lead to increased glucose and “glycation products” [48,49]. Indeed, some studies have reported increased diabetes in GW veterans [50], which would favor potential for the sucrose solution, promoting increased blood glucose and glycation products.

Improvements in these rat behavioral signs following drug treatment has been used to screen for potential antidepressant drugs in preclinical research [51,52]. In agreement with previous studies that reported antidepressant-like effects of KET in rodents [53,54,55], the present study demonstrated that, in an OP-based rat model for GWI-like depressive signs, treatment with KET rapidly decreased the FST immobility time, increased sucrose consumption on the SPT, and increased open-arm exploration of the EPM at doses that were not associated with the locomotor retardation in the OFT. Interestingly, KET’s effect on lowering FST immobility time was sustained at 24 h post-injection, but its effect on improving EPM open-arm exploration could not be observed at this timepoint.

KET possess rapid pharmacokinetic profile with a reported half-life of around 60 min in rats following an anesthetic dose [56]. Thus, it is interesting that antidepressant effects of KET are continued to be observed even when KET has been eliminated from the rat’s system. KET is a multi-modal drug and various mechanisms have been proposed for its rapid and long-lasting antidepressant action (reviewed in [53,57,58,59]). For example, GluN2B containing NMDARs are reported to be involved in the rapid antidepressant actions of KET [60]. In addition, KET-mediated NMDAR blockade at rest is also thought to be involved in its rapid antidepressant action via the deactivation eukaryotic elongation factor 2 (eEF2) kinase [61]. The activity of eEF2 kinase, also known as CamKIII, is modulated by calcium and calmodulin. We recently demonstrated that hippocampal neurons from GWI rats exhibited chronic calcium elevations [33,34]. It will be interesting to investigate the role of KET on GWI calcium homeostatic mechanisms and its subsequent effect on modulating the rapid behavioral response in this model. The fast-acting and long-lasting antidepressant effect of KET is also thought to involve augmentation of the neurotrophic factor Brain-Derived Neurotrophic Factor (BDNF) [57,58]. Ongoing studies in our laboratory are assessing the role of BDNF signaling in GWI depression and its alteration following KET administration in GWI rats.

Despite KET’s superior antidepressant efficacy, its use is limited by its psychotomimetic effects. However, recent reports suggest that the beneficial antidepressant effect of KET could be dissociated from its CNS side-effects by taking advantage of its stereoisomerism [62,63,64]. KET is a racemic mixture consisting of equal parts (*R*-)-KET and (*S*+)-KET [62,65]. While *S*-KET has 4-fold greater affinity for NMDAR compared to *R*-KET, it is the *R*-enantiomer that has been reported to possess longer lasting antidepressant effects in rodent models of depression. Indeed, in our studies, *R*-KET appeared to be more potent than *S*-KET in significantly reducing acute FST immobility time. The effects of *R*-KET were more long-lasting than *S*-KET. Further, *R*-KET’s effects on EPM behavior appeared to be stronger than KET but, like KET, this effect was not observed at 24 h. S-KET did not affect EPM behavior at either timepoint. Interestingly, it is reported that *R*-KET may produce its antidepressant effect independent of NMDAR inhibition and may involve AMPAR activation by its metabolites [63]. The role of KET metabolites in driving the antidepressant response is actively being researched [27,58]. Thus, the neurobiological mechanisms underlying the antidepressant actions of KET appear to be more complex than a simple blockade of NMDAR [66,67,68]. Alterations in cholinergic regulation by acetylcholinesterase (AChE) inhibitors including OPs is reported in the literature [9,69,70,71]. In addition, cholinergic signaling regulates glutamatergic transmission [72] including after OP exposure [69,73]. Given that OP-based GWI animal models have identified neuronal loss, elevated calcium-levels, inflammation, and reduced synaptic transmission underlying the expression of GWI neurological signs [34,73,74,75,76,77,78], there is a compelling argument that glutamatergic signaling alterations may be present in GWI, and KET, via its pleiotropic actions, could prove to be an effective treatment option for aspects of GWI.

## 5. Conclusions

Our studies have shown that KET and its enantiomers improved behavioral signs in an OP-based DFP rat model that exhibits features of GWI. These results reproduce the reported rapid and sustained actions of KET in rodent behavioral assays that are used pre-clinically to screen for antidepressant-like actions. KET’s effect was observed at low, sub-anesthetic concentrations. Enantiomeric differences in the potency and duration of the response further indicate that *R*-KET could extend the behavioral improvement profile with limited CNS side-effects. These initial studies provide a starting point for further assessment of KET in clinical trials with GWI veterans in the search for effective therapies for aspects of GWI.
